# Retinal neurovascular and mitochondrial function signatures related to cognition improvement following yoga exercise in older adults

**DOI:** 10.1371/journal.pone.0357464

**Published:** 2026-09-01

**Authors:** Mariam Moarefi, Kylie Martinez, Giovana Rosa Gameiro, Andrew Hoover, Brooke Fitzgerald, Collin Rich, Joseph Signorile, Natalie C. Ebner, Jianhua Wang, Hong Jiang

**Affiliations:** 1 Department of Ophthalmology, Bascom Palmer Eye Institute, University of Miami Miller School of Medicine, Miami, Florida, United States of America; 2 Department of Kinesiology and Sport Sciences, University of Miami, Miami, Florida, United States of America; 3 Department of Ophthalmology and Visual Sciences, Federal University of Sao Paulo – EPM/UNIFESP, Sao Paulo, Sao Paulo, Brazil; 4 Duke University School of Medicine, Durham, North Carolina, United States of America; 5 OcuSciences, Inc., Ann Arbor, Michigan, United States of America; 6 Center for Cognitive Aging and Memory, McKnight Brain Institute, University of Florida, Gainesville, Florida, United States of America; 7 Department of Psychology, University of Florida, Gainesville, Florida, United States of America; 8 Department of Neurology, University of Miami Miller School of Medicine, Miami, Florida, United States of America; University of South Florida, UNITED STATES OF AMERICA

## Abstract

**Purpose:**

To investigate the associations between retinal neurovascular and mitochondrial function signatures and cognitive performances following a 24-week yoga intervention in healthy older adults.

**Methods:**

Thirty healthy older adults (mean age 72 ± 6 years; 25 females) participated in three 60-minute sessions per week of either YogaCue or Hatha yoga over 24 weeks. Retinal structure and vessel density were measured using optical coherence tomography angiography (OCTA), while retinal blood flow (RBF) was assessed using Retinal Function Imager (RFI). Mitochondrial function was evaluated via macular flavoprotein fluorescence (FPF) using the OcuMet Beacon. Cognitive performance was assessed at baseline and follow-up using the NIH Toolbox, Trail Making Tests (TMT-A/B), and the Hopkins Verbal Learning Test (HVLT).

**Results:**

Several nominally significant correlations were observed between changes in cognitive performance and changes in retinal measures. These included associations between changes in Flanker Test performance and both RBF (ρ = 0.49, p = 0.007) and retinal capillary function (ρ = 0.42, p = 0.03); between changes in Total Recall and retinal tissue perfusion (ρ = 0.39, p = 0.04) and capillary function (ρ = 0.41, p = 0.04); and between changes in List Sorting performance and optic nerve head FPF (ρ = –0.61, p = 0.003). However, none of these associations remained statistically significant after Benjamini–Hochberg false discovery rate correction.

**Conclusions:**

This pilot study identified exploratory associations between changes in retinal neurovascular and mitochondrial function and changes in cognitive performance following a yoga intervention in healthy older adults. Although these findings may suggest potential retinal signatures of cognitive change, they should be interpreted as hypothesis-generating and require validation in larger independent cohorts before definitive conclusions can be drawn.

## Introduction

Cognitive decline is a well-documented age-related change that affects key domains such as executive function, working memory, and episodic memory, which, in turn, can compromise independence and quality of life in older adults [1,2]. As pharmacological strategies for preventing or reversing cognitive impairment remain limited, there is growing interest in lifestyle-based interventions, particularly those combining physical activity and mind-body practices, to maintain cognitive function and support cognitive resilience [3].

Yoga is a multifaceted practice that incorporates physical postures, breath regulation, and mindfulness, which has gained increased attention for its cognitive and emotional benefits in older adults [4]. Systematic reviews and meta-analyses consistently show that yoga improves key cognitive domains, including attention, memory, and processing speed [5]. Moreover, randomized controlled trials demonstrate that yoga styles, such as Hatha yoga, are not only safe and feasible for aging populations, but also yield significant improvements in executive function and mood regulation [5–7]. Despite these positive outcomes, the precise biological mechanisms through which yoga promotes cognitive health remain largely unclear and warrant further investigation.

Emerging evidence indicates that both physical exercise and mind-body practices promote neuroprotection by enhancing neuroplasticity, cerebral blood flow, and neurotrophic signaling pathways involving factors such as BDNF, IGF-1, and VEGF [8]. Yoga, in particular, may uniquely influence these mechanisms by engaging additional pathways related to autonomic nervous system regulation, stress reduction, and inflammation control [9,10]. Furthermore, mitochondrial function, central to cellular energy metabolism and brain health, is increasingly recognized as a key factor in cognitive aging and may be positively affected by movement and mindfulness-based practices [11].

The retina, as an accessible extension of the central nervous system, offers a unique opportunity to monitor microvascular, neuronal, and mitochondrial function noninvasively [12]. Advanced imaging techniques, such as optical coherence tomography angiography (OCTA) and retinal function imaging (RFI), enable the quantification of retinal tissue and vessel structure, as well as blood flow dynamics [13]. Retinal abnormalities, including reduced vessel density and thinning of the ganglion cell inner plexiform layer, have been associated with cognitive impairment and neurodegenerative conditions, highlighting the potential of retinal biomarkers as indicators of brain health [14,15]. In addition, mitochondrial function can be evaluated in the retina, providing a unique opportunity to study cell function [16].

With multimodal retinal imaging, retinal neurovascular and mitochondrial function signatures in response to physical exercises can be explored. Using these novel techniques, this study aimed to investigate the associations between retinal neurovascular and mitochondrial function signatures and cognitive performance following a 24-week yoga intervention in healthy older adults.

## Methods

### Participants

This study was approved by the University of Miami Institutional Review Board (UM IRB No. 20230735) and conducted in accordance with the Declaration of Helsinki. Written informed consent was obtained from all participants after a thorough explanation of study procedures. Recruitment began on September 29, 2023, and concluded on September 26, 2024. Thirty healthy older adults (mean age: 72 ± 6 years; 25 females) were recruited from a departmental research registry maintained by the University of Miami’s Department of Kinesiology and Sport Sciences. At enrollment, none were undergoing physical therapy, and all reported no history of neurological or psychiatric conditions. Participants’ prior physical levels were assessed; individuals who were previously sedentary were included.

Inclusion criteria required participants to be 55 years or older and report no subjective memory complaints. The MoCA was administered at baseline to characterize cognitive status but was not used as an enrollment criterion. Exclusion criteria included uncontrolled cardiovascular or neuromuscular disease, cerebrovascular conditions, HIV or other immunodeficiencies, autoimmune or inflammatory disorders (e.g., lupus, rheumatoid arthritis), a history of ocular trauma, glaucoma, diabetic retinopathy, or any other significant medical illness.

After baseline testing, participants were randomly assigned to one of two intervention groups: Hatha or YogaCue yoga. Baseline measurements were collected before the 24-week intervention, with follow-up assessments conducted within 2–3 weeks of program completion.

### Retinal imaging

Retinal assessments were performed using multiple non-invasive imaging modalities to evaluate vascular structure, blood flow, and mitochondrial function. The same eye, preferably the right, was imaged consistently across all sessions. Pupils were dilated with 1% tropicamide, and participants rested in a dimly lit room for 10 minutes before imaging.

#### Retinal blood flow.

Retinal blood flow was measured using the Retinal Function Imager (RFI; Optical Imaging Ltd., Rehovot, Israel), a fundus camera-based system that employs stroboscopic illumination and high-speed video capture to track the movement of erythrocytes within retinal vessels. This non-invasive method, which uses red blood cells as intrinsic contrast agents without the need for external dyes, has been extensively described in previous studies [13,17].

Image acquisition was synchronized with the cardiac cycle via a finger pulse sensor to reduce physiological variability. The primary field of view used was 35 degrees (7.3 × 7.3 mm^2^). At least four well-focused images per session were collected across 3–5 sessions. Vessel tracing was initially performed manually by trained investigators on a selected image using proprietary software (Browse, version 2.2.0.236). Retinal blood flow measurements were processed by the same trained investigators (MM and JW) using a standardized vessel-tracing protocol. Image analysis was not performed with formal masking to study time point (baseline versus follow-up). The software then automatically registered and tracked vessels across sessions to calculate average flow velocities. Blood flow was quantified within a 2.5 mm diameter circle centered on the fovea. Vessel diameters at the circle’s perimeter were measured from intensity profiles taken perpendicular to the vessel centerlines, using custom MATLAB software. Total arteriolar inflow and venular outflow crossing this boundary were summed and averaged to estimate macular blood flow, based on the assumption of near-equal inflow and outflow.

#### Retinal tissue volume.

Retinal tissue volume (RTV) and layer-specific measurements were obtained using a spectral-domain OCTA system (OptoVue, Inc., Fremont, CA). This non-invasive imaging modality provides high-resolution visualization of retinal structures and microvasculature. The system operates at a scanning speed of 70,000 A-scans per second with an axial resolution of 5 μm [16]. Six-by-six millimeter scans were analyzed using Orion image analysis software (Voxeleron LLC, Pleasanton, CA), which segmented six distinct intra-retinal layers and generated volumetric data. These layers included the retinal nerve fiber layer (RNFL), ganglion cell-inner plexiform layer (GCIPL), inner nuclear layer (INL), outer plexiform layer (OPL), outer nuclear layer (ONL), and photoreceptor layer (PR). RTV was evaluated within an annular region centered on the fovea, bounded by an outer diameter of 2.5 mm and an inner diameter of 0.6 mm.

#### Retinal capillary perfusion density, vessel density, and vessel length density.

Retinal vascular network (RVN) density was evaluated using the same OCTA system (AngioVue, OptoVue, Inc., Fremont, CA). For each participant, scans of 3 × 3 mm centered on the fovea were obtained. Image quality was graded on a 10-point scale, with only images scoring 7 or above included in the analysis. Following established protocols, large vessels (≥25 µm diameter) were identified and removed from the images to isolate the microvasculature, which was then skeletonized. Retinal capillary perfusion density (CPD) was then quantified within a circular region of 2.5 mm diameter centered on the fovea [13].

Retinal vessel density (RVD) and vessel length density (RVLD) were derived from the OCTA scans using en face images that met quality criteria (score ≥ 7/10). Images were resampled to 1024 × 1024 resolution and processed with custom MATLAB software. After preprocessing steps such as grayscale inversion and illumination correction, large vessels (≥25 µm) were excluded to isolate the capillary network. Remaining vessels were skeletonized, and VD and VLD were calculated within a standardized annular region (0.6–2.5 mm from the foveal center) using ImageJ. VD represented the area fraction occupied by vessels, while VLD reflected the total vessel length per area unit (mm^−1^) [18].

#### Retinal tissue perfusion and capillary function.

Retinal tissue perfusion (RTP) was calculated by dividing RBF within a 2.5 mm diameter circle centered on the fovea by the corresponding inner RTV, which included the inner four retinal layers: retinal nerve fiber layer (RNFL), ganglion cell–inner plexiform layer (GCIPL), inner nuclear layer (INL), and outer plexiform layer (OPL). This measure reflects the average perfusion rate per mm^3^ of inner retinal tissue [17]. Retinal capillary function (RCF) was calculated as the ratio of RBF to retinal vessel length density (RVLD), representing the average flow per millimeter of capillary length [18].

#### Choriocapillaris density and foveal avascular zone assessment.

Choriocapillaris density was assessed using OCTA scans (AngioVue, OptoVue, Fremont, CA) acquired over a 3 × 3 mm region centered on the macula. The choriocapillaris layer was automatically segmented from 9 μm above to 31 μm below Bruch’s membrane. Flow density, defined as the percentage of the area showing perfusion, was calculated within a 2.5 mm-diameter circle centered on the fovea using the device’s built-in software. Scans with a signal strength index below 7/10 or with motion or segmentation artifacts were excluded from analysis [19].

Foveal avascular zone (FAZ) metrics were also derived from the same OCTA scans. The AngioVue software automatically delineated the FAZ in en face retinal vascular network slabs, with manual corrections performed by trained graders when needed. The primary outcome was FAZ area (mm^2^).

#### Retinal mitochondrial function.

Retinal mitochondrial function was evaluated using flavoprotein fluorescence (FPF) imaging with the OcuMet Beacon (OcuSciences, Ann Arbor, MI), a method previously described in the literature [16]. This technique measures the green autofluorescence emitted by oxidized mitochondrial flavoproteins upon blue-light excitation, serving as a marker of oxidative stress and mitochondrial activity [20,21]. Imaging was performed at two retinal locations: the foveal pit and the optic nerve head (ONH).

The acquisition process included capturing an infrared fundus image over a 60° × 21.5° field of view, followed by flavoprotein fluorescence measurement within a 17° × 21.5° region centered on the macula and ONH. Lens signal compensation was applied based on patient age and intraocular lens status to adjust measurements. The device uses infrared (825–870 nm) and blue (458 ± 2 nm) LEDs for illumination, detecting autofluorescence between 520 and 540 nm. Optical filters are employed to enhance the signal-to-noise ratio and minimize interference from other retinal fluorophores [22]. Image quality was assessed using the OcuMet RMA software (version 3.0.2), with exclusion criteria including poor image quality (e.g., shading artifacts or inadequate focus), off-center alignment, missing data, or pupillary diameter below 3.5 mm.

#### Cognitive function assessment.

Cognitive performance was evaluated using a validated neuropsychological battery encompassing multiple domains of fluid cognition, memory, and executive function. The NIH Toolbox Cognition Battery was used to assess core aspects of cognitive functioning. The Dimensional Change Card Sort Test (DCCS) measured cognitive flexibility and set-shifting, key components of executive function [23]. The Flanker Inhibitory Control and Attention Test assessed selective attention and inhibitory control, reflecting the ability to suppress irrelevant information and maintain task focus [23]. Episodic memory was evaluated using the Picture Sequence Memory Test, which required participants to recall the correct temporal order of a series of pictured events [24].

Working memory was assessed with the List Sorting Working Memory Test, a sequencing task involving both visual and auditory stimuli. Processing speed was measured using the Pattern Comparison Processing Speed Test, which required participants to rapidly determine whether two visual patterns were the same or different [23]. Raw scores from each test were analyzed individually, along with the Fluid Cognition Composite Score (FCS), which provides an aggregate measure of performance across fluid cognitive domains.

Verbal learning and memory were assessed using the Hopkins Verbal Learning Test–Revised (HVLT-R), a widely used instrument for measuring verbal recall and recognition [25]. The HVLT-R includes trials of immediate recall, delayed recall, and recognition discrimination. The following subcomponents were analyzed independently: total recall, delayed recall, learning slope, retention percentage, and recognition discrimination index.

Executive function, cognitive flexibility, visual scanning, and psychomotor speed were further assessed using the Trail Making Test (TMT), parts A and B. TMT-A required participants to sequentially connect numbers (1–25). At the same time, TMT-B involved alternating between numbers and letters in ascending order. The time taken to complete each task served as the primary outcome variable, with longer durations indicating poorer performance [26].

#### Yoga intervention.

All subjects attended either Hatha or YogaCue classes for three 60-minute sessions per week over a 24-week period. Attendance was monitored throughout the 24-week intervention period. Participants were required to attend at least 85% of scheduled yoga sessions to be considered compliant with the intervention protocol and eligible for inclusion in the final analyses. Only participants who completed both baseline and follow-up retinal and cognitive assessments were included in the study analyses.

Both yoga styles featured standing poses commonly associated with Hatha yoga, including Warrior Poses 1 and 2 (Virabhadrasana 1 and 2), Mountain Pose (Tadasana), Triangle Pose (Trikonasana), Tree Pose (Vrikshasana), and Airplane Pose (Dehasana). Each session began with a warmup, transitioned into the main sequence, and concluded with a cooldown and passive resting postures.

The YogaCue program incorporated two major modifications over the traditional Hatha yoga: reduced recovery times between poses and the integration of visual and auditory cues to guide the practice. This approach focused on cue-based neuromotor-cognitive integration, distinguishing it from traditional Hatha Yoga, which emphasized classic postures, breathing techniques, and relaxation. Certified instructors led both programs.

Cognitive assessments and retinal imaging were conducted by separate research teams, with cognitive testing performed before retinal imaging. Most retinal imaging sessions were performed during the morning or early afternoon hours. Participants remained seated in the examination environment for at least 15 minutes before image acquisition.

### Statistical analysis

All statistical analyses were conducted using SPSS version 31.0 (SPSS Inc., Chicago, IL, USA). Data normality was evaluated using the Shapiro–Wilk test. Continuous variables are presented as mean ± standard deviation (SD). Change scores (Δ = follow-up − baseline) were calculated for all retinal and cognitive parameters. Given that the primary objective was to examine associations between retinal and cognitive changes across the intervention cohort, participants from the Hatha and YogaCue groups were pooled for the primary analyses. Baseline characteristics and intervention-related change scores were compared between groups using Mann–Whitney U tests for continuous variables and Fisher’s exact test for sex. Spearman’s rank correlation coefficients (ρ) were calculated to assess associations between changes in cognitive outcomes and changes in retinal measures.

Outliers were identified using standardized z-scores calculated separately for each change-score variable. Observations with an absolute z-score greater than 3 (|z| > 3) were considered statistical outliers and excluded from the corresponding correlation analyses. Statistical significance was set at p < 0.05. To account for multiple comparisons, the Benjamini–Hochberg false discovery rate (FDR) procedure was applied across all retinal-cognitive correlations presented in Table 2, and corresponding q-values were calculated.

No a priori sample size calculation was performed for the retinal-cognitive correlation analyses because this study represents a secondary exploratory analysis of a previously completed intervention cohort. The sample size was therefore determined by participant availability and completion of the parent study.

The cohort included only five male participants; consequently, the study was neither designed nor sufficiently powered to evaluate sex-specific retinal-cognitive associations. Sex was therefore not included as a covariate in the primary exploratory correlation analyses.

The primary objective was to investigate associations between changes in retinal biomarkers and changes in cognitive performance during a yoga intervention. Accordingly, participants from the YogaCue and Hatha groups were pooled for the primary analyses. Given that each intervention arm included only 15 participants, the study was not adequately powered to detect differences between yoga modalities. Combining both groups increased statistical power for evaluating retinal-cognitive associations across the intervention cohort.

## Results

Thirty participants were enrolled in the study, with a mean age of 72 ± 6 years (range: 56–84 years, 25 females; Table 1).

**Table 1:** Characteristics of study participants.

Variables	Mean ± SD	Range
Sex (5 M/25 F)		
Age (y)	72 ± 6	56–84
Height (cm)	163.7 ± 8.5	154–191
Weight (kg)	75.2 ± 15.6	49.7–108.7
BMI (kg ⋅ m^−2^)	28.0 ± 5.0	19.9–36.7
Systolic BP	126.4 ± 14.2	91–158
Diastolic BP	76.9 ± 7.9	60–90
MOCA	27.3 ± 2.3	20–30
IOP	13.7 ± 1.6	11–17

BMI = Body Mass Index; BP = Blood Pressure; MOCA = Montreal Cognitive Assessment; IOP = Intraocular Pressure.

For FPF analyses, 5 macular FPF measurements and 8 ONH FPF measurements were excluded because of missing data or failure to meet predefined image-quality criteria. Across all change-score variables included in the correlation analyses, 18 outlier instances met the predefined statistical outlier criterion (|z| > 3).

Participants demonstrated high adherence to the intervention, with a mean attendance rate of 91% across both yoga groups. All included participants satisfied the predefined attendance requirement of at least 85% of scheduled sessions. To examine potential differences between the two yoga interventions, participants were randomly assigned in a stratified manner to either the YogaCue group (n = 15; 12 females; mean age = 73 ± 7 years) or the Hatha yoga group (n = 15; 13 females; mean age = 72 ± 5 years).

Comparisons between the Hatha and YogaCue groups demonstrated no significant differences in age or retinal imaging measures at baseline (S1 Table). Cognitive performance was also comparable between groups for nearly all outcomes, with the exception of the Discrimination Index (p = 0.043). Importantly, no significant between-group differences were identified in intervention-related changes in retinal or cognitive outcomes (all p > 0.05; S1 Table).

**Fig 1:**
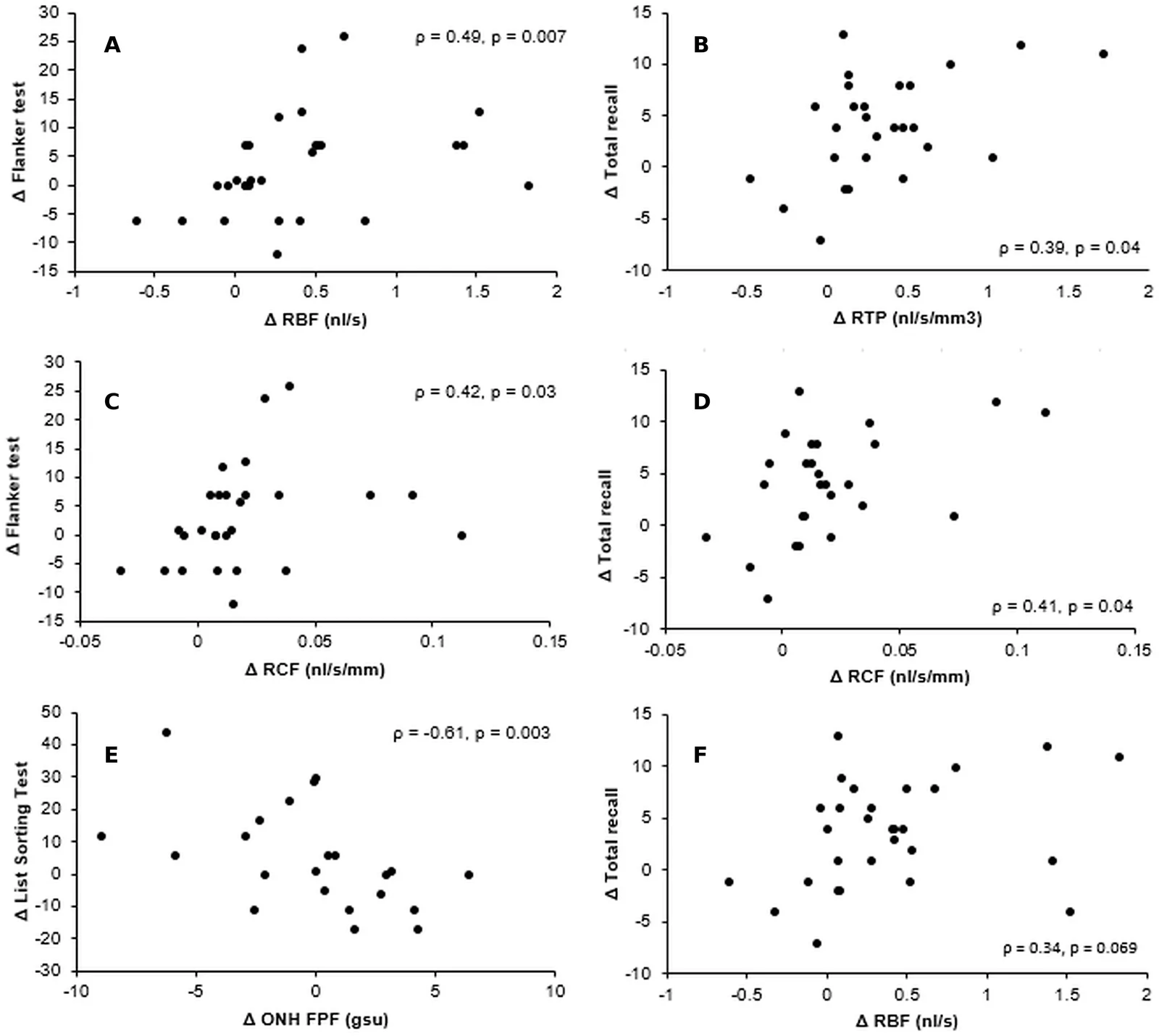
Relations between changes in cognitive tests and changes in retinal parameters. Scatterplots show the relationships between (A) the Flanker Test with retinal blood flow, (B) total recall with retinal tissue perfusion, (C) list sorting test with optical nerve head flavoprotein fluorescence, (D) total recall with retinal capillary function, (E) Flanker test with retinal capillary function, and (F) total recall with retinal blood flow. RBF = retinal blood flow; RTP = retinal tissue perfusion; RCF = retinal capillary function; ONH FPF = optical nerve head flavoprotein fluorescence.

Correlations were analyzed between pre- to post-intervention changes in cognitive outcomes and pre- to post-intervention changes in retinal parameters after removing outliers. Significant positive correlations were observed between pre-to post-intervention changes in the Flanker Test and the pre- to post-intervention changes in both RBF (ρ = 0.49, p = 0.007, Table 2, Fig 1) and RCF (ρ = 0.42, p = 0.03); and between changes in HVLT-R Total Recall and changes in RTP (ρ = 0.39, p = 0.04) and in RCF (ρ = 0.41, p = 0.04). In contrast, changes in the List Sorting Test were negatively correlated with changes in ONH FPF (ρ = −0.61, p = 0.003). Although not significant, a trend was noted between changes in Total Recall and changes in RBF (ρ = 0.34, p = 0.07).

**Table 2:** Spearman correlation coefficients between pre- to post-intervention changes in cognitive test scores and pre- to post-intervention changes in retinal parameters without outliers.

	Δ RBF	Δ RTP	Δ RCF	Δ Mac FPF	Δ ONH FPF	Δ RVD	Δ RVLD	Δ RVN	Δ CCD	Δ FAZ	Δ RTV
**Δ Dimensional Change Card Sort**	0.01	−0.10	−0.06	0.08	0.38	−0.02	0.14	0.28	0.13	0.28	−0.02
**Δ Flanker Test**	.487**	0.38	.422*	−0.12	0.04	0.15	0.03	0.08	−0.13	−0.20	0.10
**Δ Picture Sequence Memory Test**	−0.14	−0.07	−0.04	0.27	0.04	−0.20	−0.09	−0.01	0.17	0.00	0.30
**Δ List Sorting Test**	−0.06	−0.06	−0.10	−0.06	−.611**	−0.19	−0.19	−0.28	−0.12	−0.07	0.18
**Δ Pattern Comparison**	−0.24	−0.23	−0.13	−0.22	0.00	−0.16	−0.09	0.09	0.03	−0.29	0.09
**Δ Fluid Cognition Composite Score**	0.19	0.14	0.21	0.13	−0.23	−0.16	0.01	0.17	0.12	−0.09	0.18
**Δ Trail Making A**	0.23	0.26	0.30	0.20	−0.07	−0.16	−0.23	−0.11	−0.03	−0.09	−0.02
**Δ Trail Making B Seconds**	−0.01	−0.04	0.05	−0.03	−0.18	0.18	−0.10	0.03	−0.35	0.07	−0.23
**Δ Total Recall**	0.34	.392*	.405*	0.37	0.02	−0.12	−0.18	−0.03	0.16	0.10	0.10
**Δ Delayed Recall**	0.20	0.10	0.10	0.23	0.23	0.06	0.14	0.01	0.16	0.03	0.08
**Δ Percent Retained**	0.06	−0.03	−0.04	0.11	0.37	0.15	0.23	0.04	0.06	−0.05	0.09
**Δ True Positive**	0.01	0.08	0.07	0.17	−0.16	0.14	−0.19	−0.11	−0.27	−0.11	0.01
**Δ False Related**	0.18	0.10	0.01	0.04	0.08	0.05	0.23	0.35	−0.19	−0.33	0.16
**Δ False Unrelated**	0.02	0.05	0.02	0.14	−0.02	0.21	−0.01	−0.04	−0.15	−0.01	−0.24
**Δ Discrimination Index**	0.03	0.16	0.12	−0.02	−0.35	0.03	−0.18	−0.23	0.00	−0.05	−0.15

RBF = retinal blood flow; RTP = retinal tissue perfusion; RCF = retinal capillary function; Mac FPF = macular flavoprotein fluorescence; ONH FPF = optical nerve head flavoprotein fluorescence; RVD = vessel density; RVLD = vessel length density; CCD = choriocapillaris density; FAZ = foveal avascular zone. Note: bold font indicates statistical significance. * Correlation is significant at the 0.05 level (2-tailed), ** Correlation is significant at the 0.01 level (2-tailed). Nominal p-values are shown. Benjamini–Hochberg false discovery rate–adjusted q-values are presented in S2 Table.

Given the large number of retinal-cognitive correlations examined, Benjamini–Hochberg false discovery rate correction was applied across all correlations presented in Table 2. Although five retinal-cognitive associations reached nominal statistical significance (p < 0.05), none remained statistically significant after FDR correction (all q > 0.05; S2 Table).

Sensitivity analyses comparing correlations with and without outlier exclusion are presented in S3 Table. The associations between Total Recall and retinal hemodynamic parameters (RTP and RCF), as well as the association between List Sorting performance and ONH FPF, remained largely unchanged. The association between Flanker performance and RCF was attenuated when the outlier was retained, whereas the association between Flanker performance and RBF remained statistically significant in both analyses.

## Discussion

This study investigated associations between changes in cognitive performance and changes in retinal vascular and mitochondrial function observed during a 24-week yoga intervention in healthy older adults. The primary objective was to explore whether retinal biomarkers and cognitive measures changed in parallel during the intervention period. Therefore, the findings should be interpreted as exploratory associations between within-person change trajectories rather than evidence of intervention efficacy. While prior research has shown that yoga can improve cognitive performance [27], and recent findings in patients with Parkinson’s disease have shown a correlation between retinal microvascular changes and cognitive gains [28], the present study is the first to explore this relationship in a cognitively unimpaired older adult sample. Improvements in a subset of cognitive measures were associated with changes in retinal circulatory parameters and mitochondrial function, suggesting that the retina may provide a noninvasive window into systemic or cerebral vascular and functional adaptations linked to cognitive health.

However, these findings should be interpreted with caution, as significant correlations were observed in only a few cognitive tests relative to the total number assessed. Importantly, none of the observed associations remained significant after correction for multiple comparisons; therefore, the biological interpretations discussed below should be considered exploratory.

It is well established that the retina shares embryological origin, anatomical features, and physiological properties with the brain, including similar blood-retina and blood-brain barriers and comparable microvascular architecture [29]. Due to this homology, the retina is increasingly recognized as a potential surrogate for cerebral changes, particularly in the context of aging and neurodegenerative conditions [30–32]. Most prior studies, however, have examined cognitive or retinal measures in isolation. The present findings extend this literature by suggesting that changes in retinal perfusion, rather than structural measures alone, may parallel improvements in specific cognitive domains, particularly verbal memory and executive function, in older adults. These results are consistent with the hypothesis that yoga-related enhancements in cognitive performance could be partly mediated by functional vascular adaptations that are detectable in the retina.

Our results do not directly suggest a causal effect for the retina in cognitive enhancement; rather, it is more likely that retinal and cerebral tissues respond similarly to systemic or localized vascular influences [33]. The observed associations suggest that retinal blood flow may reflect broader neurovascular processes occurring in the brain [34]. In alignment with this proposition, previous research has shown that cerebral hypoperfusion can serve as an early marker of cognitive decline, often preceding detectable structural brain changes [35–37]. Likewise, impairments in retinal perfusion have been observed in individuals with mild cognitive impairment and Alzheimer’s disease [17,38,39]. These parallels support the emerging view of the retina as an accessible, noninvasive window to the brain, with potential applications in early diagnosis and monitoring of cognitive health across the lifespan and in aging.

Among the various retinal parameters evaluated, we found that circulation-related indices, such as RBF, RTP, and RCF, were associated with cognitive changes. In contrast, vessel structure-related parameters, such as RVN, VD, and VLD, were not. This may reflect differences in temporal sensitivity among these biomarkers: blood flow can respond rapidly to physiological demands, whereas structural vascular remodeling typically occurs over more extended periods or under pathological conditions, making it less likely to be detected after the six-month intervention [40,41]. Retinal tissue perfusion, which normalizes flow to tissue volume, provides a dynamic index of metabolic supply-demand balance and may be particularly informative in capturing early functional changes relevant to cognition in aging or neurodegenerative disease [17]. While the number of significant associations was limited, the observed pattern aligns with the hypothesis that functional retinal vascular measures are more sensitive to short-term cognitive or metabolic adaptations than structural metrics.

In addition to vascular measures, ONH FPF showed a significant association with cognitive change in our study; specifically, a strong correlation with the List Sorting Test, a task indexing working memory and executive function [23]. ONH FPF reflects mitochondrial oxidative stress and metabolic dysfunction in neural tissue, particularly in retinal ganglion cells [42,43]. The selective association with the List Sorting Test may reflect the particular sensitivity of working memory and executive processes to metabolic and neurovascular adaptations induced by yoga, although other cognitive domains may also benefit without being detectable in this small sample.

The inclusion of ONH FPF in the present study expands the scope of retinal biomarkers compared to previous work, providing a more integrative framework in which vascular and metabolic signals from the retina jointly reflect broader brain health in aging. While ONH FPF showed significance, macular FPF was not significantly associated. One possible explanation is that the ONH region, which contains unmyelinated axons and dense glial and vascular networks, may be more metabolically sensitive to neurovascular changes that accompany aging, whereas macular FPF predominantly reflects photoreceptor and retinal pigment epithelium metabolism that may be less directly linked to brain-wide neurodegenerative processes. Future studies may benefit from incorporating ONH FPF alongside perfusion measures to capture a more comprehensive view of neurovascular metabolic resilience.

The integrative retinal pattern observed in the present study aligns with findings in cerebral physiology, where regional cerebral blood flow is closely tied to cognitive performance and has been shown responsive to interventions such as aerobic exercise and mindfulness-based practices [44,45]. Blood flow alterations in the retina may, therefore, represent a parallel response to such interventions, offering a practical means to monitor training efficacy. Notably, increased retinal blood flow without concurrent changes in vessel density suggests an upregulation of functional perfusion within existing capillary networks, possibly indicating an early adaptive response to increased cognitive or metabolic demand [46].

This interpretation is consistent with prior findings that cognitive improvements can be mediated by multiple, parallel mechanisms, including but not limited to improved vascular function [47,48]. While retinal imaging provides valuable insight into the vascular dimension, it cannot fully capture neural, hormonal, or synaptic processes also implicated in cognitive change. Moreover, some participants demonstrated cognitive improvement without significant changes in retinal parameters, suggesting that the retina, though a valuable proxy, cannot entirely substitute for direct assessment of cognition and brain function [34]. Nevertheless, given its accessibility, non-invasive nature, and emerging technological precision, retinal imaging represents a promising adjunctive tool for monitoring brain health, particularly when neuroimaging is not feasible or cost-effective [12].

Some limitations of this study should be acknowledged. First, the sample size was determined by the yoga intervention study, and no a priori power calculation was performed for the retinal-cognitive correlation analyses. Consequently, these analyses should be considered exploratory and hypothesis-generating. Although significant associations were identified, the study may have been underpowered to detect smaller retinal-cognitive relationships, and nonsignificant findings should therefore be interpreted with caution. In addition, a large number of retinal-cognitive correlations were examined. Although five associations reached nominal statistical significance, none remained significant after Benjamini–Hochberg false discovery rate correction. Therefore, these findings should be interpreted as exploratory and hypothesis-generating until replicated in larger independent cohorts.

In addition, the cohort was predominantly female (25 of 30 participants, 83%), which may limit the generalizability of the findings and may have reduced the ability to detect potential sex-specific differences in retinal vascular physiology, cognitive aging trajectories, and responses to yoga intervention. Future studies with larger and more balanced cohorts are needed to determine whether the observed retinal-cognitive associations differ by sex.

Second, participants from the YogaCue and Hatha groups were pooled for the primary analyses. Although supplementary analyses revealed no significant differences in intervention-related retinal or cognitive changes between groups (S1 Table), pooling may have obscured subtle modality-specific effects. Future studies with larger sample sizes specifically designed to compare yoga modalities will be necessary to determine whether different forms of yoga produce distinct retinal or cognitive responses.

Third, although the follow-up period was substantial for behavioral interventions, it may still have been too short to capture slower, structural changes in the retinal vasculature. The cohort consisted of generally healthy older adults with relatively high baseline cognitive function, which may limit the generalizability of the results to clinical or more diverse populations. In addition, assessment timing, caffeine intake, recent physical activity before study visits, and the interval between the most recent yoga session and retinal imaging were not standardized, which may have contributed to variability in retinal vascular measurements.

Finally, the absence of a non-exercising control group represents an important limitation. Consequently, it is not possible to determine whether the observed retinal and cognitive changes were specifically related to the intervention or reflected practice effects, regression to the mean, natural temporal variation, or other non-specific factors. Therefore, the present findings should be interpreted as exploratory associations between within-person changes in retinal and cognitive measures rather than evidence of intervention efficacy. Furthermore, despite assessing multiple cognitive domains, the study design does not allow conclusions regarding the causal direction of the observed associations. Future studies incorporating sedentary control groups and alternative exercise interventions will be necessary to determine the specificity and underlying biological basis of the observed retinal-cognitive associations.

## Conclusion

This pilot study is the first to examine associations between changes in retinal neurovascular and mitochondrial function and cognitive performance following a yoga intervention in healthy older adults. The findings provide preliminary evidence that changes in specific cognitive domains were associated with changes in retinal perfusion and mitochondrial activity during the intervention period, supporting the potential of retinal imaging as a non-invasive approach for investigating neurovascular and metabolic processes related to cognitive health.

However, because the study lacked a non-exercising control group and involved multiple exploratory analyses, these findings should be considered hypothesis-generating rather than evidence of intervention efficacy. Future studies incorporating sedentary control groups, alternative exercise interventions, and larger longitudinal cohorts will be necessary to determine the specificity and underlying biological basis of the observed retinal-cognitive associations and to evaluate whether these retinal signatures may serve as reliable biomarkers of cognitive change in aging populations.

## Supporting information

S1 Table Comparison of baseline characteristics and intervention-related changes between the Hatha and YogaCue groups. (DOCX)

S2 Table Retinal–cognitive correlations reaching nominal significance before and after Benjamini–Hochberg correction. (DOCX)

S3 Table Sensitivity analysis of significant retinal–cognitive associations. (DOCX)
