# Supplementary material for: Retinal neurovascular and mitochondrial function signatures related to cognition improvement following yoga exercise in older adults
Source: PLoS One. 2026 Sep 1;21(9):e0357464. doi: 10.1371/journal.pone.0357464 (PMC13533345; doi:10.1371/journal.pone.0357464)
Supplement: Supplementary file 1 [file pone.0357464.s001.docx]

| **S1 Table.** Comparison of baseline characteristics and intervention-related changes between the Hatha and YogaCue groups. | | | |
| --- | --- | --- | --- |
|  |  |  |  |
| **Variable** | **Hatha** | **YogaCue** | **p-value** |
| **Baseline characteristics** |  |  |  |
| Female sex, n (%) | 12 (80%) | 13 (86.7%) | 1.00 |
| Age (years) | 73.1 ± 6.8 | 71.5 ± 5.2 | 0.22 |
| **Retinal outcomes (baseline)** |  |  |  |
| Macular FPF | 32.4 ± 11.9 | 32.6 ± 9.6 | 0.47 |
| ONH FPF | 37.7 ± 14.0 | 36.7 ± 6.7 | 0.95 |
| Retinal Blood Flow (RBF) | 2.22 ± 0.41 | 2.21 ± 0.54 | 0.66 |
| Retinal Capillary Function (RCF) | 0.118 ± 0.021 | 0.115 ± 0.030 | 0.38 |
| Retinal Tissue Perfusion (RTP) | 1.90 ± 0.40 | 1.92 ± 0.67 | 0.43 |
| **Cognitive outcomes (baseline)** |  |  |  |
| Dimensional Change Card Sort | 123.5 ± 17.0 | 123.4 ± 15.0 | 0.87 |
| Flanker Test | 110.4 ± 12.1 | 105.9 ± 10.7 | 0.31 |
| Picture Sequence Memory Test | 111.5 ± 12.1 | 104.2 ± 16.8 | 0.33 |
| List Sorting Test | 105.9 ± 14.8 | 106.6 ± 10.4 | 0.80 |
| Pattern Comparison | 102.6 ± 24.4 | 103.1 ± 20.0 | 0.72 |
| Fluid Cognition Composite Score | 114.1 ± 15.1 | 111.5 ± 10.1 | 0.88 |
| Trail Making Test A | 30.7 ± 11.1 | 27.7 ± 8.8 | 0.55 |
| Trail Making Test B (seconds) | 77.9 ± 51.1 | 69.5 ± 28.0 | 0.66 |
| Total Recall | 21.6 ± 5.3 | 24.2 ± 3.5 | 0.16 |
| Delayed Recall | 6.2 ± 3.7 | 6.4 ± 3.4 | 0.85 |
| Percent Retained | 63.9 ± 34.1 | 61.5 ± 29.3 | 0.56 |
| True Positive | 10.6 ± 1.7 | 11.5 ± 0.9 | 0.07 |
| False Related | 1.8 ± 1.7 | 1.1 ± 1.3 | 0.28 |
| False Unrelated | 0.13 ± 0.35 | 0.13 ± 0.52 | 0.61 |
| Discrimination Index | 8.67 ± 2.82 | 10.20 ± 1.52 | 0.04 |
| **Change scores (Δ = Follow-up − Baseline)** |  |  |  |
| Δ Macular FPF | -0.82 ± 2.31 | -0.32 ± 8.41 | 0.98 |
| Δ ONH FPF | -0.67 ± 3.67 | 0.25 ± 3.86 | 0.58 |
| Δ RBF | 0.37 ± 0.60 | 0.63 ± 1.10 | 0.66 |
| Δ RCF | 0.022 ± 0.038 | 0.031 ± 0.057 | 0.84 |
| Δ RTP | 0.39 ± 0.55 | 0.54 ± 1.05 | 0.98 |
| Δ Dimensional Change Card Sort | 2.0 ± 11.3 | 4.1 ± 17.1 | 0.53 |
| Δ Flanker Test | 2.73 ± 8.54 | 3.40 ± 9.28 | 0.74 |
| Δ Picture Sequence Memory Test | -1.47 ± 15.21 | 3.80 ± 18.59 | 0.57 |
| Δ List Sorting Test | 5.13 ± 14.58 | 4.60 ± 14.61 | 0.80 |
| Δ Pattern Comparison | 7.53 ± 15.60 | 2.87 ± 11.86 | 0.60 |
| Δ Fluid Cognition Composite Score | 6.73 ± 11.62 | 7.13 ± 9.80 | 0.66 |
| Δ Trail Making Test A | 0.62 ± 10.50 | -2.38 ± 5.97 | 0.72 |
| Δ Trail Making Test B (seconds) | -9.90 ± 39.96 | -1.09 ± 35.46 | 0.63 |
| Δ Total Recall | 5.33 ± 4.79 | 2.00 ± 4.93 | 0.10 |
| Δ Delayed Recall | 2.13 ± 1.85 | 1.07 ± 3.35 | 0.25 |
| Δ Percent Retained | 14.19 ± 18.15 | 9.42 ± 30.66 | 0.49 |
| Δ True Positive | 0.27 ± 1.79 | -0.27 ± 1.10 | 0.41 |
| Δ False Related | -0.53 ± 1.41 | 0.13 ± 1.25 | 0.29 |
| Δ False Unrelated | 0.00 ± 0.38 | -0.07 ± 0.26 | 0.58 |
| Δ Discrimination Index | 0.80 ± 2.54 | -0.33 ± 1.45 | 0.20 |
| Values are presented as mean ± standard deviation unless otherwise indicated. Between-group comparisons were performed using Mann–Whitney U tests for continuous variables and Fisher's exact test for sex. | | | |
