# Supplementary material for: Retinal neurovascular and mitochondrial function signatures related to cognition improvement following yoga exercise in older adults
Source: PLoS One. 2026 Sep 1;21(9):e0357464. doi: 10.1371/journal.pone.0357464 (PMC13533345; doi:10.1371/journal.pone.0357464)
Supplement: Supplementary file 2 [file pone.0357464.s002.docx]

| **S2 Table.** Retinal–cognitive correlations reaching nominal significance before and after Benjamini–Hochberg correction | | | | |
| --- | --- | --- | --- | --- |
| **Cognitive outcome** | **Retinal parameter** | **ρ** | **p** | **q** |
| ΔFlanker Test | ΔRBF | 0.487 | 0.007 | 0.539 |
| ΔFlanker Test | ΔRCF | 0.422 | 0.028 | 0.907 |
| ΔList Sorting Test | ΔONH FPF | -0.611 | 0.003 | 0.462 |
| ΔTotal Recall | ΔRCF | 0.405 | 0.036 | 0.907 |
| ΔTotal Recall | ΔRTP | 0.392 | 0.043 | 0.907 |

RBF = retinal blood flow; RTP = retinal tissue perfusion; RCF = retinal capillary function; ONH FPF = optic nerve head flavoprotein fluorescence. Δ denotes the change from baseline to follow-up (follow-up − baseline). Spearman correlation coefficients (ρ) are reported.
