# Supplementary material for: Retinal neurovascular and mitochondrial function signatures related to cognition improvement following yoga exercise in older adults
Source: PLoS One. 2026 Sep 1;21(9):e0357464. doi: 10.1371/journal.pone.0357464 (PMC13533345; doi:10.1371/journal.pone.0357464)
Supplement: Supplementary file 3 [file pone.0357464.s003.docx]

| **S3 Table.** Sensitivity analysis of significant retinal-cognitive associations | | | |  |  |  |  |
| --- | --- | --- | --- | --- | --- | --- | --- |
| **Cognitive Outcome** | **Retinal Parameter** | **N (With Outliers)** | **ρ (With Outliers)** | **p** | **N (Without Outliers)** | **ρ (Without Outliers)** | **p** |
| Δ Flanker Test | Δ RBF | 30 | 0.375 | **0.04** | 29 | 0.487 | **0.01** |
| Δ Flanker Test | Δ RCF | 28 | 0.311 | 0.11 | 27 | 0.422 | **0.03** |
| Δ Total Recall | Δ RTP | 28 | 0.396 | **0.04** | 27 | 0.392 | **0.04** |
| Δ Total Recall | Δ RCF | 28 | 0.408 | **0.03** | 27 | 0.405 | **0.04** |
| Δ List Sorting Test | Δ ONH FPF | 22 | -0.611 | **0.003** | 22 | -0.611 | **0.003** |

RBF = retinal blood flow; RTP = retinal tissue perfusion; RCF = retinal capillary function; ONH FPF = optic nerve head flavoprotein fluorescence. Δ denotes the change from baseline to follow-up (follow-up − baseline). Spearman correlation coefficients (ρ) are reported.
